# The impact of social activities and physical exercise on arterial stiffness (AIP) among older Chinese adults from CHARLS

**DOI:** 10.1097/MD.0000000000043654

**Published:** 2025-08-15

**Authors:** Cheng-ming Zhong, An-yang Liu

**Affiliations:** a Department of General Surgery, School of Clinical Medicine, Tsinghua Medicine, Tsinghua University, Beijing Tsinghua Changgung Hospital, Beijing, China.

**Keywords:** atherogenic index of plasma (AIP), cardiovascular risk, older adults, sedentary behavior, sex differences, vigorous physical activity

## Abstract

The atherogenic index of plasma (AIP), a biomarker reflecting lipid metabolism and atherosclerotic risk, is increasingly recognized as a critical indicator in cardiovascular health. However, the influence of social activities and physical exercise on AIP, particularly with respect to sex-specific differences, remains underexplored. This study examined these associations using data from 3278 participants aged 60 years or older in the 2015 wave of the China Health and Retirement Longitudinal Study. Results showed that sedentary social activities, such as playing Mahjong or chess, were significantly associated with elevated AIP levels, particularly in females (β = 0.043, *P* < .05). In contrast, vigorous physical activity significantly reduced AIP levels in both males (β = −0.089, *P* < .001) and females (β = −0.065, *P* < .01), with stronger effects observed in males. Moderate and light physical activities showed no significant associations with AIP. These findings highlight the sex-specific associations between social and physical activities and AIP levels in older adults. Public health interventions should encourage dynamic social engagement and vigorous physical exercise, tailored to gender differences, to reduce cardiovascular risk and promote arterial health.

## 1. Introduction

Atherosclerosis is a chronic inflammatory condition characterized by lipid accumulation in the vascular intima, infiltration of inflammatory cells, and reduced arterial elasticity, ultimately leading to cardiovascular disease (CVD).^[1]^ The Atherogenic Index of Plasma (AIP), calculated as the logarithm of the triglycerides to high-density lipoprotein cholesterol ratio (log [triglycerides/HDL-C]), has emerged as a robust biomarker for assessing dyslipidemia, lipid metabolism status, and atherosclerosis risk.^[2,3]^ Elevated AIP values are strongly associated with an increased risk of cardiovascular events, particularly in individuals with metabolic syndrome, diabetes, or hypertension.^[4,5]^ Moreover, AIP demonstrates high sensitivity and specificity in evaluating atherosclerosis progression, assisting clinicians in stratifying cardiovascular risk and formulating personalized interventions.^[3,6]^

Beyond its role in cardiovascular risk assessment, AIP has been increasingly utilized in predicting and managing metabolic syndrome, obesity, and related disorders.^[7,8]^ Given its clinical significance, understanding the modifiable factors influencing AIP is essential for developing effective public health strategies aimed at reducing the incidence of CVD. Among such factors, lifestyle behaviors, including physical activity and social engagement, are particularly noteworthy.^[9]^ Physical activity is widely recognized as a cornerstone of cardiovascular health promotion. Regular exercise improves lipid profiles, reduces inflammation, and lowers the risk of atherosclerosis.^[10–12]^ However, modern sedentary lifestyles – characterized by leisure activities such as watching television, playing computer games, and prolonged sitting – contribute to physical inactivity and increased obesity rates, exacerbating cardiovascular risks.^[13]^ Conversely, engaging in vigorous physical activities, such as aerobic exercise and resistance training, has been shown to lower AIP levels and improve cardiovascular outcomes.^[12]^ Despite extensive evidence on the cardiovascular benefits of physical exercise, the independent and interactive effects of different activity intensities on AIP remain underexplored, particularly with respect to gender-specific differences.^[10,11]^

Similarly, social activities play an important role in shaping health outcomes, yet their association with cardiovascular biomarkers such as AIP is less well understood.^[14,15]^ While social engagement is linked to improved mental health and reduced mortality risk,^[16]^ some sedentary social activities, such as playing Mahjong or chess, may contribute to prolonged inactivity, unhealthy dietary habits, and stress. These behaviors could adversely affect lipid metabolism and increase cardiovascular risk, especially among females, who may exhibit greater sensitivity to environmental and psychological factors. However, evidence on the gender-specific effects of social activities on AIP is limited.

This study seeks to address these gaps by investigating the relationships between social activities, physical exercise, and AIP levels in older Chinese adults, with a focus on sex-specific differences. Using data from the nationally representative China Health and Retirement Longitudinal Study (CHARLS), this study integrates lifestyle behaviors and biological outcomes to provide novel insights into the modifiable factors influencing AIP. The findings aim to inform gender-tailored public health interventions and contribute to the broader understanding of cardiovascular risk reduction strategies in aging populations.

## 2. Methods

### 2.1. Study design and participants

This study utilized data from the CHARLS, a nationally representative longitudinal survey initiated in 2011, with follow-ups conducted every 2 years. CHARLS aims to collect comprehensive data on the health, economic circumstances, and social conditions of individuals aged 45 years and older in China. The survey was implemented by the Chinese Center for Disease Control and Prevention (CCDCP) and covered 28 provinces and 150 counties across China. Data collection procedures included in-home interviews, physical examinations, and other assessments, capturing information on health status, demographics, socioeconomic factors, and physiological measurements. Detailed descriptions of the CHARLS methodology are available on the official website (https://charls.pku.edu.cn/) and in prior publications.^[17]^

**Figure 1.**
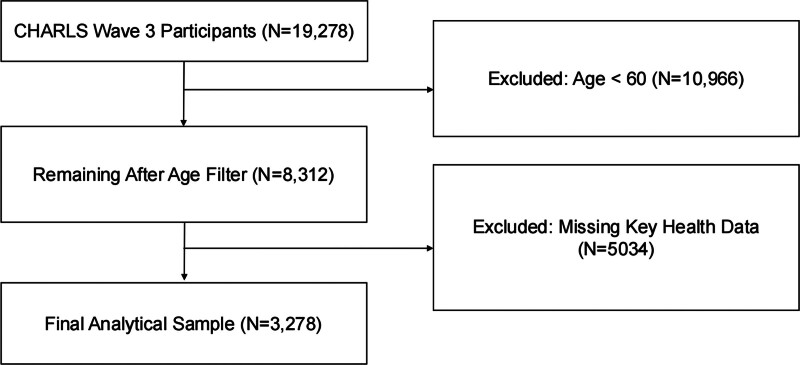
Flowchart of participant selection.

For this analysis, data from the 2015 wave of CHARLS were utilized. The original dataset included 19,278 participants. Participants were excluded if they were younger than 60 years old, had missing gender data, or lacked key health information such as social activity participation, exercise intensity, or lipid profiles. After applying these exclusion criteria, a total of 3278 eligible participants were retained for the final analysis. The detailed process of participant selection is illustrated in a flowchart (Fig. 1).

### 2.2. Data collection and covariates

In the CHARLS survey, data were collected through structured questionnaires administered by trained interviewers. These questionnaires captured detailed information on participants’ marital status, behavioral habits, and medical history. Fasting venous blood samples were collected, centrifuged, and stored at −20°C before being transported to the CCDCP in Beijing for laboratory analysis within 2 weeks. Laboratory assessments included measurements of triglycerides (TG), high-density lipoprotein cholesterol (HDL-C), and other relevant biomarkers.

Key variables used in the analysis included demographic characteristics, health indicators, social activities, and physical activity. Demographic variables consisted of age, gender, educational attainment, residential area (rural or urban), and body mass index (BMI). Health indicators included baseline HDL-C (bl_hdl), triglycerides (bl_tg), and fasting blood glucose (bl_glu).

Social activity variables captured the frequency of engaging in the following activities: visiting friends or participating in social interactions (social1), playing games such as Mahjong or cards (social2), providing unpaid assistance to relatives or neighbors (social3), participating in group activities such as dancing or exercising (social4), involvement in social organizations (social5), and volunteering or engaging in charitable activities (social6).

Physical activity variables included the frequency of vigorous (vgact_c), moderate (mdact_c), and light (ltact_c) physical activities, representing varying levels of exercise intensity. These variables provided a comprehensive overview of participants’ lifestyle behaviors and their potential impact on cardiovascular health.

### 2.3. Statistical analysis

Descriptive statistics were calculated to summarize the demographic, health, and behavioral characteristics of the study population. Continuous variables were reported as means ± standard deviations, while categorical variables were presented as frequencies and percentages. A baseline table was constructed to provide an overview of these variables across the study population.

The primary outcome, AIP, was categorized into quartiles to allow for group comparisons. Multivariable linear regression analyses were employed to examine the associations between social activities, physical exercise, and AIP. These analyses were stratified by gender to explore potential sex-specific differences, with adjustments made for age, BMI, residential area, and other relevant covariates to minimize confounding effects.

All statistical analyses were conducted using R software (version 4.2.0). Statistical significance was set at a 2-tailed *P*-value of < .05.

The baseline characteristics of the study participants, stratified by quartiles of the AIP, are presented in Table 1. Significant differences were observed across groups in demographic and clinical variables. Participants in the highest AIP quartile (level 4) were younger (67.11 ± 5.93 years) compared to those in the lowest quartile (level 1: 68.25 ± 6.62 years, *P* < .001). The proportion of males progressively declined from level 1 (57%) to level 4 (44%, *P* < .001), while higher education levels were more common in higher quartiles (*P* = .006). The proportion of rural residents decreased significantly from level 1 (72%) to Level 4 (56%, *P* < .001). Clinically, HDL-C levels were highest in level 1 (62.95 ± 12.80 mg/dL) and lowest in level 4 (42.66 ± 7.43 mg/dL, *P* < .001), while triglycerides and fasting glucose levels showed significant increases across quartiles (*P* < .001).

**Table 1:** Baseline characteristics of the study population across quartiles of AIP.

	Level				
	1	2	3	4	*P*-test
n	820	820	819	819	–
Age (mean [SD])	68.25 (6.62)	68.35 (6.49)	67.96 (6.42)	67.11 (5.93)	<.001
Gender (mean [SD])	0.57 (0.50)	0.51 (0.50)	0.47 (0.50)	0.44 (0.50)	<.001
Edu (mean [SD])	1.66 (0.88)	1.68 (0.89)	1.70 (0.93)	1.81 (0.98)	.006
Rural (mean [SD])	0.72 (0.45)	0.66 (0.47)	0.59 (0.49)	0.56 (0.50)	<.001
BMI (mean [SD])	23.12 (28.09)	24.24 (24.24)	24.72 (10.86)	25.54 (7.41)	.097
bl_hdl (mean [SD])	62.95 (12.80)	52.90 (9.24)	48.29 (8.31)	42.66 (7.43)	<.001
bl_tg (mean [SD])	70.38 (15.51)	98.97 (18.99)	137.57 (28.74)	253.37 (97.90)	<.001
bl_glu (mean [SD])	97.51 (29.32)	99.26 (23.41)	105.59 (33.14)	117.80 (48.20)	<.001
social1 (mean [SD])	0.31 (0.46)	0.35 (0.48)	0.34 (0.47)	0.37 (0.48)	.081
social2 (mean [SD])	0.15 (0.35)	0.17 (0.38)	0.20 (0.40)	0.21 (0.41)	.001
social3 (mean [SD])	0.13 (0.34)	0.14 (0.35)	0.13 (0.34)	0.12 (0.32)	.566
social4 (mean [SD])	0.05 (0.21)	0.05 (0.23)	0.06 (0.24)	0.09 (0.28)	.003
social5 (mean [SD])	0.02 (0.15)	0.02 (0.15)	0.02 (0.13)	0.03 (0.18)	.149
social6 (mean [SD])	0.01 (0.09)	0.01 (0.09)	0.01 (0.09)	0.02 (0.14)	.042
vgact_c (mean [SD])	0.40 (0.49)	0.33 (0.47)	0.27 (0.44)	0.24 (0.43)	<.001
mdact_c (mean [SD])	0.53 (0.50)	0.49 (0.50)	0.47 (0.50)	0.50 (0.50)	.181
ltact_c (mean [SD])	0.79 (0.41)	0.79 (0.41)	0.76 (0.42)	0.81 (0.39)	.135

AIP = atherogenic index of plasma.

Behavioral variables also demonstrated notable differences. Vigorous physical activity levels declined steadily from level 1 (0.40 ± 0.49) to level 4 (0.24 ± 0.43, *P* < .001), whereas moderate and light physical activities showed no significant differences across groups. Regarding social activities, significant variations were observed in playing games such as Mahjong (social2), group exercise (social4), and volunteering (social6, *P* < .05), while other activities (social3 and social5) showed no significant differences. These results highlight the interplay between demographic, clinical, and lifestyle factors with AIP levels, underscoring the importance of tailored interventions to address cardiovascular risk factors in older adults.

## 3. Results

A linear regression analysis was performed to investigate the associations between 6 types of social activities (social1–social6) and the AIP, adjusting for potential confounding factors. The results are summarized in Table 2.

**Table 2:** Associations between social activities and AIP: multivariable regression results.

Social_activity	Term	Estimate	Std. error	Statistic	*P*-value
social1	(Intercept)	0.373005644	0.006009108	62.07337712	0
social1	social1	0.020486851	0.010248317	1.999045444	.045685908
social2	(Intercept)	0.372121096	0.005378012	69.19306219	0
social2	social2	0.043386027	0.012580932	3.448554352	.000570705
social3	(Intercept)	0.382840782	0.005218208	73.36633683	0
social3	social3	−0.021582313	0.014509181	-1.487493517	.136980755
social4	(Intercept)	0.376406753	0.00502494	74.90771565	0
social4	social4	0.05796042	0.0200448	2.891544027	.003858781
social5	(Intercept)	0.379030176	0.004929918	76.88366276	0
social5	social5	0.04175332	0.031557254	1.323097381	.185895334
social6	(Intercept)	0.378957998	0.004893426	77.44226705	0
social6	social6	0.102196182	0.047356922	2.1579988	.031000376

AIP = atherogenic index of plasma.

The findings revealed significant positive associations for certain social activities. Specifically, social1 (interaction with friends) was positively associated with AIP (estimate = 0.0205, *P* = .0457), indicating that increased interaction with friends corresponded to elevated AIP levels. Similarly, social2 (participation in community activities) demonstrated a significant positive relationship with AIP (estimate = 0.0434, *P* = .0006), suggesting that frequent community engagement was linked to higher AIP levels. For social4 (group activities or exercise), a significant positive association was also observed (estimate = 0.0579, *P* = .0039), highlighting the potential influence of group-based activities on AIP.

In contrast, social3 (visiting relatives and friends) and social5 (voluntary work) did not exhibit statistically significant relationships with AIP (*P* > .05). Notably, social6 (participation in social clubs or organizations) showed a strong positive association with AIP (estimate = 0.1022, *P* = .031), implying its potential impact on lipid metabolism and cardiovascular risk.

These results suggest that specific social activities, particularly community participation, group-based activities, and involvement in social organizations, are positively associated with AIP levels. This may reflect a possible link between social engagement and cardiovascular health. However, the underlying mechanisms remain unclear. Future research should further explore these associations, including the potential influence of gender differences on the observed relationships (Table 3).

**Table 3:** Gender-specific regression analysis of physical activity and AIP.

Gender	Term	Estimate	Std. error	Statistic	*P*-value
Female	Age	−0.00304	0.00159	−1.9	.0572
Male	Age	−0.00579	0.00159	−3.65	.000276
Female	ltact_c	−0.00459	0.0241	−0.19	.849
Male	ltact_c	0.0325	0.0255	1.27	.203
Female	mdact_c	−0.000389	0.0207	−0.0188	.985
Male	mdact_c	−0.00403	0.0212	−0.19	.849
Female	vgact_c	−0.0653	0.0232	−2.81	.0051
Male	vgact_c	−0.0895	0.0224	−4	6.96 × 10^−05^

AIP = atherogenic index of plasma.

**Figure 2.**
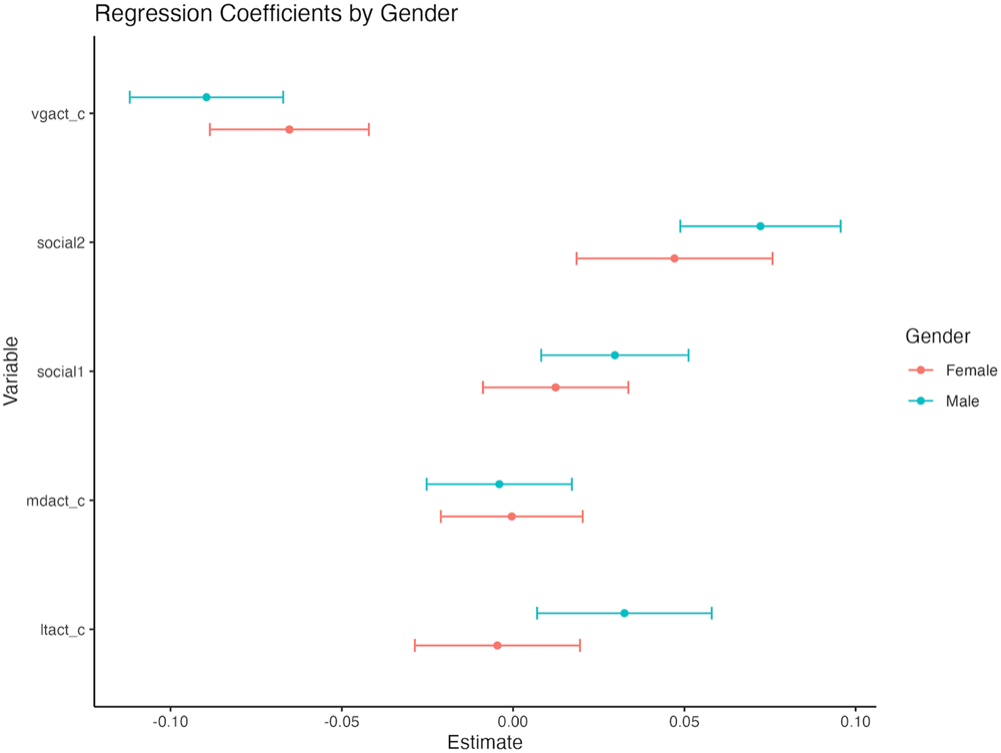
Gender-specific regression coefficients for social and physical activities on AIP. AIP = atherogenic index of plasma.

The gender-stratified analysis revealed notable differences in the relationship between age, physical activity, and AIP. In males, age showed a stronger and statistically significant negative association with AIP (estimate = −0.00579, *P* = .0003), whereas in females, the association was weaker and not statistically significant (estimate = −0.00304, *P* = .0572). These findings suggest that age may have a greater impact on AIP reduction in males compared to females (Fig. 2).

Regarding physical activity, vigorous physical activity (vgact_c) exhibited a significant negative association with AIP in both genders, with a stronger effect observed in males (estimate = −0.0895, *P* < .001) compared to females (estimate = −0.0653, *P* = .0051). In contrast, light (ltact_c) and moderate-intensity physical activity (mdact_c) were not significantly associated with AIP in either gender (*P* > .05), indicating a limited effect of lower-intensity activities.

These findings highlight the critical role of vigorous physical activity in reducing AIP, particularly among males, and its potential as a key intervention for mitigating cardiovascular risk. Furthermore, the results underscore the importance of developing tailored strategies that address gender differences in the influence of age and physical activity on cardiovascular health.

The regression analysis highlights significant gender differences in the effects of physical activity and social participation on AIP. Vigorous physical activity (vgact_c) demonstrates a significant negative association for both males and females, indicating its protective role in reducing AIP levels. The effect, however, is more pronounced in males, as reflected by a larger negative estimate and narrower confidence intervals, suggesting that men may gain greater cardiovascular benefits from high-intensity physical activity.

In contrast, social participation (social2) shows a positive association with AIP for both genders, with males exhibiting slightly higher estimates, indicating a potentially stronger influence of social engagement on AIP in men. Light (ltact_c) and moderate (mdact_c) physical activities, on the other hand, exhibit minimal or nonsignificant associations with AIP in both genders, suggesting limited contributions of lower-intensity activities to AIP reduction.

These findings emphasize the gender-specific effects of physical activity and social participation, underscoring the importance of designing tailored interventions that consider these differences to optimize cardiovascular health outcomes.

## 4. Discussion

This study, based on the CHARLS dataset, systematically explored the impact of social activities and physical activity on the AIP, uncovering significant gender-specific differences. The results revealed that sedentary social activities were associated with elevated AIP levels, particularly among females, while vigorous physical activity demonstrated a protective effect in both sexes, with males deriving more pronounced benefits. These findings expand the existing knowledge base on AIP risk factors and provide valuable theoretical insights for developing personalized cardiovascular disease prevention strategies.

### 4.1. The role of social activities: a complex relationship

One of the key contributions of this study is its systematic evaluation of different social activity types and their effects on AIP,^[18]^ with a focus on gender-specific differences. Sedentary social activities, such as playing Mahjong or board games, were positively associated with higher AIP levels, likely due to prolonged sitting, unhealthy dietary habits, and psychological stress.^[19]^ This effect was particularly pronounced in females, potentially reflecting sex-based physiological and psychological differences.^[20]^ This research suggests that females are more prone to environmental and emotional factors in social contexts, such as increased calorie consumption and sedentary behavior, which exacerbate lipid metabolism disorders. It is important to note that not all social activities are detrimental. Dynamic social activities have been shown to improve psychological resilience, promote physical activity, and enhance overall health outcomes.^[21]^ Public health interventions should therefore aim to reduce the duration of sedentary social activities while encouraging more interactive and physically engaging forms of social participation.

### 4.2. Protective effects of vigorous physical activity

Vigorous physical activity was strongly associated with lower AIP levels, reaffirming its critical role in preventing atherosclerosis. High-intensity activities, such as running and high-intensity interval training,^[11]^ were found to significantly improve AIP levels in both sexes, with more pronounced benefits observed in males. These effects can be attributed to mechanisms such as increased energy expenditure, enhanced fat oxidation, and reduced chronic inflammation. The greater improvements in AIP among males are likely due to higher muscle mass and metabolic rates, consistent with existing literature.

In females, the benefits of vigorous exercise may be moderated by factors such as hormonal fluctuations, which could dampen metabolic responses to intense activity. Nonetheless, females can achieve meaningful reductions in AIP by incorporating strength training with aerobic exercise, highlighting the importance of tailored exercise programs to maximize cardiovascular benefits across genders.

### 4.3. Sex-specific mechanisms and public health implications

This study emphasizes the biological and psychological factors underlying gender-specific differences in AIP-related behaviors. Females appeared more sensitive to sedentary behaviors, potentially leading to higher fat storage and elevated AIP levels. Additionally, the emotional burdens associated with familial or societal roles may exacerbate cardiovascular risks in females during sedentary social activities. Conversely, males showed more significant improvements in insulin sensitivity and inflammation reduction during vigorous exercise, facilitating greater AIP improvements. These findings underscore the need for targeted public health strategies. For females, efforts should focus on reducing sedentary activity durations and promoting dynamic social activities, while for males, encouraging participation in vigorous exercise programs will likely yield the greatest cardiovascular benefits. Tailored interventions addressing sex-specific behaviors are critical for mitigating cardiovascular risks effectively.

### 4.4. Research contributions and novelty

The novelty of this study lies in its integration of social and physical activities’ effects on AIP, combined with an exploration of sex-specific differences. Unlike prior research focusing on single activity types, this study employed multivariable analyses to reveal the independent and interactive effects of diverse activities on AIP. The use of the nationally representative CHARLS dataset enhances the generalizability of these findings and provides a robust foundation for developing evidence-based health policies.

### 4.5. Limitations and future directions

Despite offering valuable insights, this study has limitations. First, the reliance on self-reported data introduces the potential for recall bias. Second, the study quantified social activities based solely on frequency without considering their quality or context. Third, the cross-sectional design limits causal inference. Future studies should incorporate objective measurements, such as wearable devices, to improve accuracy and adopt longitudinal designs to explore the dynamic effects of lifestyle factors on AIP. Additionally, further research should investigate the underlying mechanisms driving the observed gender-specific differences.

### 4.6. Policy implications

The findings of this study provide actionable insights for public health interventions. Reducing sedentary social activities among older adults, particularly high-risk females, should be a priority. Governments and communities should promote dynamic social engagements and develop gender-specific exercise programs. High-intensity exercise plans should be encouraged for males, while females may benefit from combining moderate-intensity exercise with dynamic social activities. Public health campaigns emphasizing the importance of AIP and its relationship with cardiovascular risks are essential for raising awareness and reducing the global burden of cardiovascular diseases.

## 5. Conclusions

This study, based on the CHARLS dataset, provides new evidence on the associations between social activities, physical activity, and AIP, emphasizing significant gender-specific differences. The findings demonstrate that:

1. Social activities: sedentary social activities, such as Mahjong or board games, are positively associated with elevated AIP levels, particularly among females. In contrast, dynamic social activities, like group exercise or dancing, show potential protective effects on AIP.
2. Physical activity: vigorous physical activity significantly reduces AIP levels in both genders, with males exhibiting more pronounced benefits. Light and moderate physical activities, however, have minimal or nonsignificant effects on AIP.
3. Gender-specific differences: females are more susceptible to the adverse effects of sedentary behavior, while males derive greater cardiovascular benefits from high-intensity physical activity.

These findings highlight the importance of designing tailored, gender-specific public health interventions. For females, reducing sedentary social activity durations and promoting dynamic social engagements are key strategies, whereas for males, high-intensity exercise programs should be prioritized. By addressing these gender-specific behaviors, targeted interventions can effectively mitigate cardiovascular risks and improve overall health outcomes.

Future research should adopt longitudinal designs and incorporate objective measurements to further elucidate the mechanisms underlying these associations and validate causal relationships. Public health efforts should also raise awareness of AIP as an important cardiovascular risk marker, promoting lifestyle changes to reduce the burden of cardiovascular diseases.

## Author contributions

**Conceptualization:** Cheng-ming Zhong, An-yang Liu.

**Data curation:** Cheng-ming Zhong.

**Formal analysis:** Cheng-ming Zhong.

**Investigation:** Cheng-ming Zhong.

**Software:** Cheng-ming Zhong.

**Supervision:** Cheng-ming Zhong.

**Writing – original draft:** Cheng-ming Zhong.

**Writing – review & editing:** Cheng-ming Zhong.
